# Neovascular Progression and Retinal Dysfunction in the Laser-Induced Choroidal Neovascularization Mouse Model

**DOI:** 10.3390/biomedicines11092445

**Published:** 2023-09-02

**Authors:** Anna Salas, Anna Badia, Laura Fontrodona, Miguel Zapata, José García-Arumí, Anna Duarri

**Affiliations:** 1 Ophthalmology Research Group, Vall d’Hebron Institut de Recerca, 08035 Barcelona, Spain; 2 Department of Ophthalmology, Vall d’Hebron Hospital Universitari, 08035 Barcelona, Spain

**Keywords:** age-related macular degeneration, neovascular, laser-induced choroidal neovascularization, retina, fibrosis

## Abstract

The mouse model of laser-induced choroidal neovascularization (LI-CNV) has been widely used to study neovascular age-related macular degeneration; however, it still lacks a comprehensive characterization. Here, CNV was induced in the eyes of 12-week-old C57BL/6J male mice by argon laser irradiation. We studied the CNV lesion progression of an LI-CNV mouse cohort by using multimodal imaging (color fundus, optical coherence tomography (OCT), and fluorescence angiography, focal electroretinography features for 14 days, and related cytokines, angiogenic factors, and reactive gliosis for 5 days. CNV lesions involving the rupture of the Bruch’s membrane were confirmed using funduscopy and OCT after laser photocoagulation. During the initial stage, from the CNV induction until day 7, CNV lesions presented leakage observed by using fluorescence angiography and a typical hyperreflective area with cell infiltration, subretinal leakage, and degeneration of photoreceptors observed through OCT. This correlated with decreased retinal responses to light. Moreover, inflammatory and angiogenic markers were reduced to basal levels in the first 5 days of CNV progression. In contrast, reactive gliosis and the VEGF expression in retinal sections were sustained, with infiltration of endothelial cells in the subretinal space. In the second stage, between days 7 and 14 post-induction, we observed stabilization of the CNV lesions, a hyperfluorescent area corresponding to the formation of fibrosis, and a partial rescue of retinal function. These findings suggest that the LI-CNV lesion development goes through an acute phase during the first seven days following induction, and then the CNV lesion stabilizes. According to these results, this model is suitable for screening anti-inflammatory and anti-angiogenic drugs in the early stages of LI-CNV. At the same time, it is more convenient for screening anti-fibrotic compounds in the later stages.

## 1. Introduction

Age-related macular degeneration (AMD) is the leading cause of blindness in elderly over 65 years in industrialized countries and affects millions worldwide [1,2,3]. AMD can be classified into dry AMD, which accounts for 80 to 90% of the cases, and wet AMD, which is present in only 10 to 20% of the patients [4]. The wet (exudative or neovascular) form is a late stage, the most aggressive and rapidly progressive condition, and the major cause of vision loss. The abnormal blood vessels grow from the choriocapillaris through Bruch’s membrane into the subretinal space [5]. This process is known as choroidal neovascularization (CNV) or macular neovascularization and produces severe damage in the retina with fluid extravasation and photoreceptor death [6]. Several eye conditions, such as macular degeneration, myopia, histoplasmosis, angioid streaks, tumors, and traumatic and idiopathic conditions, are also linked to CNV [7].

The CNV is a pathological complex process controlled by diverse factors that regulate several steps. The CNV formation process involves blood vessel formation, cell adhesion, and migration to vessels sprouting under the retina [8]. The new vessels often lead to the rupture of the Bruch’s membrane, exudation of fluid in the subretinal space, subretinal fibrosis, hemorrhages, and hypoxia [9]. The pathological mechanisms underlying CNV involve the overproduction of vascular endothelial growth factor (VEGF) [10] and an increase in hypoxia, oxidative stress, and inflammation, which induce the activation of hypoxia-inducible factor-1 (HIF-1) [11]. Current therapy to treat wet AMD seeks to block the production of VEGF through the periodic intravitreal injection of anti-VEGF [12]. Still, novel treatments are under investigation, which explore other potential targets like HIF1-α, PDGF, TGFβ, FGF, etc. [12,13]. This is because 10% of patients do not react to anti-VEGF, and 50% of those who receive long-term treatment experience considerable vision loss within 5 years, primarily due to atrophy and the onset of subretinal fibrosis [14,15]. Subretinal fibrosis is the pathological mechanism by which fibroblasts or myofibroblasts release extracellular matrix proteins, including collagen, fibronectin, and laminin, in response to CNV damage [16]. However, pathological and transcriptome evidence suggests that CNV occurs concurrently with wound-healing responses like fibrosis [17]. Indeed, neovascular as well as extravascular components, including fibrotic tissue, RPE cells, fibroblasts, myofibroblasts, and inflammatory cells, are present in lesions associated with nAMD [18,19].

The study of cellular and molecular mechanisms of CNV has been of high interest in the last decade. Because in vitro endothelial cell culture models lack complex in vivo cellular interactions within the different retinal cell types, experimental animal models have become essential for understanding the pathogenesis of ocular neovascularization and testing novel anti-angiogenic drugs. Among all of the CNV animal models available [20], the laser-induced CNV model (LI-CNV) has become the most used to study wet AMD. The model consists of the disruption of Bruch’s membrane by laser photocoagulation of the choroid, which later induces the growth of new choroidal blood vessels into the subretinal area. This model was first employed in monkeys in 1979 [21] and has also been described and widely used in non-human primates [21,22,23], mice [24,25], rats [26,27], rabbits [28], and pigs [29,30]. Other techniques for producing AMD-like phenotypes in mice include injecting pro-angiogenic agents into the eye, such as recombinant viral vectors overexpressing VEGF [31], macrophages, lipid hydroxy peroxide, and polyethylene glycol, or injecting subretinal matrigel or beads [20]. However, none of these treatments are as effective as targeted laser injury, which caused CNV in 31% of injections in C57BL/6 mice, compared to a 70% success rate in the laser photocoagulation method in the same strain of mice [32]. Although the LI-CNV model cannot mimic the complexity of human pathology, which is strongly dependent on age, environmental factors, and genetics, it offers many other advantages, as it recapitulates the biological processes involving inflammation and angiogenesis and is relatively rapid to develop.

Remarkably, the mouse model has become the most well-established model adaptation because of the appropriate time course of events, high reliability, cost-effectiveness, and the availability of transgenic animals that permit the exploration of the molecular mechanisms during the process of CNV. However, several factors have been identified to affect the CNV evolution in this model, such as the strain, gender, and age of the animals [33,34,35,36,37]. Furthermore, although several authors widely use this model, it still lacks a complete characterization. In fact, contradictory results have been published regarding some physiopathological events, such as the time point of maximal CNV growth or the expression of inflammatory and angiogenic markers. Regarding the time point of maximal CNV growth, some studies have reported peak CNV growth occurring at 7–10 days post-laser induction, while others have reported peak growth occurring at 14–21 days post-induction [24,38,39]. Additionally, other studies have reported a biphasic growth pattern, with an initial growth phase followed by regression and a second growth phase [37]. Similarly, there are discrepancies in the expression of inflammatory and angiogenic markers in the LI-CNV model, such as the increased or unchanged expression of VEGF, TNF-α, and IL-6 or macrophage markers in the retina following laser induction [36,40,41]. Moreover, clinical translation may be hampered by the frequent use of only healthy male animals, and reproducibility is put at risk by poor reporting [42,43]. It is appealing to define and put into practice standardized reporting and methodological standards.

Here, we recruited an LI-CNV mouse cohort to study the occurrence, multimodal imaging, and focal electroretinography (ERG) features and related inflammation and fibrosis involved in the CNV lesion progression for two weeks.

## 2. Materials and Methods

### 2.1. Animals

Male C57BL/6J mice of 12 weeks (Charles River Laboratories. Saint Germain Nuelles, France) were used for all experiments and were maintained on a 12 h light/dark cycle with ad libitum food and water. All procedures were conducted under general anesthesia with a mixture of 2% isoflurane (Arrane^®^. Baxter laboratories, Victoria, Australia)/1% O_2_ using an induction camera and a rodent mask. Body temperature was maintained with a heating pad. Pupils were dilated with Tropicamide eyedrops (Colorcusi Tropicamida^®^ 10 mg/mL, Alcon Laboratories, El Masnou, Spain), and 2% Methocel gel (OmniVision, Puchheim, Germany) was administered to the cornea to favor contact with the lens.

### 2.2. Laser Photocoagulation

The choroidal neovascularization was induced in mice’s retinas *(n* = 30 LI-CNV mice and *n* = 6 control mice) by applying a green laser to rupture Bruch’s membrane as described [44,45,46,47,48]. Briefly, once the animal was anesthetized, pupils were dilated with Tropicamide eyedrops (and the animal was placed in the Micron III platform (Phoenix Research Labs., Pleasanton, CA, USA), putting the lens in contact with the cornea. Bruch’s membrane rupture was achieved by applying an argon laser of 352 nm, a power of 250 mW during 100 ms, and a fixed diameter of 50 µm by using the image-guided laser system. Animals received 4 burns per eye at clock positions 3, 6, 9, and 12 equidistant from the optic nerve.

### 2.3. Multimodal Imaging and Focal Electroretinography Recording

Animals were subjected to fundus fluorescence angiography (FA) at days 2, 4, 7, 9, 11, and 14 post-laser. Once the animals were anesthetized and pupils dilated, 10 µL/g sodium fluorescein (0.1% Fluorescein Oculos, Thea Laboratories, S.A., Spain) was administered intraperitoneally. Eye fundus images from the central retina were taken 1 min after fluorescein administration using a green filter. Leaking areas in the lasered spots were quantified using the ImageJ software [49].

Animals were placed in the Micron IV-OCT2 platform, and optical coherence tomography (OCT) with the guidance of a bright-field live fundus image was performed using the image-guided OCT system. OCT images were taken from the same lesions during the laser day and on days 2, 5, 7, and 14 post-laser. OCT images were analyzed using InSight 3D software (Voxeleron, Austin, TX, USA. Version 2.0.5807).

To assess the retinal function, retinas were examined at 7 and 14 days post-laser by using focal electroretinography (fERG), which allows the measurement of the visual response limited to particular areas. Mice were dark-adapted for 16 h, and ERG was recorded under dim red light. Animals were connected to the ERG by three electrodes: the ground electrode was placed in the tail, the reference electrode was settled subcutaneously in the head, and the recording electrode was located in contact with the cornea through the lens. Dim red light was used to situate a spot of ~2 mm in the desired area of the retina by using a live fundus image. ERG recordings were performed by stimulating the retina in the selected zones with flashes at different light intensities (200, 800, and 12,800 cd·s·m^−2^). Five to ten ERG traces were obtained for each condition, and the results were averaged. The amplitude and the implicit time of a- and b-waves were calculated using the Labscribe2 software (Phoenix Research Labs. Pleasanton, CA, USA. Version 3.013000).

### 2.4. Retinal Pigment Epithelium, Choroid, and Sclera Flat-Mounts

After euthanasia with CO_2_, eyes were enucleated and placed on ice-cold phosphate buffer saline (PBS) (Lonza. Basel, Switzerland). Under a dissecting microscope, the anterior segment, lens, and neural retina were removed, and the remaining eyecups were fixed for 20 min in methanol at −20 °C. Then, tissues were washed twice with PBS—Tween 0.1% (PBS-T) (Sigma-Aldrich. Madrid, Spain) and blocked with 2% bovine serum albumin (BSA) (Sigma Aldrich) in PBS-T for 1 h, followed by overnight incubation with FITC-conjugated Isolectin-B4 (1:50 in PBS-T + 3% BSA) at 4 °C. Finally, 5 washes for 10 min with PBS-T were performed, and the eyecups were flattened through radial cuts and mounted on glass slides with FluoroshieldTM medium (Sigma-Aldrich) [50]. The images of the lesions were taken under a fluorescence microscope (Olympus Fluoview FV1000. Hamburg, Germany). ImageJ was used to compute the fluorescence intensity region (in the green Channel, average grey level) within the maximal boundary of each CNV lesion; regions in which vasculature or optic nerve overlapped the green area were dismissed. The maximal boundary of each green-stained CNV lesion was defined using digital magnification and the ‘freehand selection tool’, and the area was measured in µm^2^.

### 2.5. Real-Time PCR

Animals were sacrificed, and eyes were enucleated and dissected under the microscope, removing the anterior segment and lens. The posterior poles (neural retina, EPR, choroid, and sclera) were snap-frozen, and three freezing–thawing cycles were performed to disrupt all of the tissues. A total of 200 µL of TrizolTM reagent (Thermo Fisher Scientific. Waltham, MA USA)) was added per sample, and RNA extraction was performed following the manufacturer’s instructions. Double-stranded cDNA was reverse-transcribed using random primers and a retrotranscription kit (Applied Biosystems. Foster City, CA, USA). Gene expression was analyzed by using real-time PCR for the genes Tnf-a (Fw: 5′-ATTCGAGTGACAAGCCTGTAG-3′, Rev: 5′-GGTTGTCTTTGAGATCCATGC-3′), mcp-1 (Fw: 5′-AATGGGTCCAGAAGTACATTAGAAA-3′, Rev: 5′- GGTGCTGAAGTCCTTAGGGTTG-3′), gfap (Fw: 5′-CGCTGGAGGAGGAGATCCA-3′. Rev: 5′- ACATCCATCTCCACGTGGACC-3′), vegf-120 (Fw: 5′-ACTGGACCCTGGCTTTACTG-3′, Rev: 5′-TCTGCTCTCCTTCTGTCGTG-3′), vegf-165 (Fw: 5′-ACAGAACAAAGCCAGAAAATCAC-3′, Rev: 5′-TCGCCTTGCAACGCGAGTC-3′), and pigf (Fw: 5′-AGCGACAAGGAACAGAACG-3′, Rev: 5′-AACTAATCTCCACACCAGCAC-3′), using a SYBR green-based PCR reaction mixture (Roche Diagnostics, SL. Sant Cugat del Vallès, Spain), programmed with a 10 min initial hot-start activation of Taq polymerase at 95 °C followed by 40 cycles of amplification (95 °C, for 10 s, 60 °C for 5 s, and 72 °C for 10 s) in a LihtCycler equipment (Roche Diagnostics, SL. Sant Cugat del Vallès, Spain). Ct values were normalized to the ß2-microglobulin (B2M) housekeeping gene and expressed as 2-ddCt.

### 2.6. Histology and Immunohistochemistry

Mice were perfused with 4% paraformaldehyde (Electron Microscopy Science. Hatfield, PA, USA), and eyes were prepared and embedded in paraffin. Tissue blocks were serially sectioned in the vertical pupillary-optic nerve plane at 4 µm thickness. Laser spots were identified within alternate sections with hematoxylin and eosin staining. Consecutive sections were used for immunohistochemistry against collagen type IV (ab6586, Abcam. Cambridge, UK), RPE-65 (SC-73616, Santa Cruz Biotech, Dallas, TX, USA), VEGF (07-1376, Millipore. Temecula, CA, USA), and GFAP (ab7260, Abcam). Paraffin microsections were first warmed at 65 °C for 1 h, and then serial washes of 3 min (2 in xylene, 2 in 100% ethanol, 1 in 96% ethanol, 1 in 70% ethanol, and 1 in 50% ethanol) were performed for the deparaffinization. Samples were then fixed and permeabilized with Methanol/acetic acid at −20 °C for 1 min, followed by 3 washes with PBS. Antigen retrieval was performed by boiling the samples embedded in citrate buffer pH 6 in a pressure cooker. Hereafter, retinal sections were blocked with 5% normal goat serum (Abcam), 5% BSA, and 0.3% Triton X in PBS for 1 h at room temperature. Primary antibodies were then applied to the samples diluted 1/500 and incubated overnight at 4 °C. Samples were then incubated with fluorescent-labeled secondary antibodies (Alexa Fluor^®^ 568 Goat Anti-Rabbit IgG (H + L) or Alexa Fluor^®^ 488 Goat anti-mouse IgG (H + L)) for 1 h at room temperature. Finally, slides were washed and mounted with FluoroshieldTM medium. Images were taken in the confocal microscope (Olympus Fluoview FV1000. Hamburg, Germany).

### 2.7. Exclusion Criteria

The images were blindfolded before the following predetermined exclusion criteria were used. Overburnt lesions, hemorrhages, and merged lesions were excluded using OCT, FA, and flat mounts.

### 2.8. Statistics

Statistical analyses were performed using commercially available software (Graph-Pad-Prism^®^ version 6.01, La Jolla, CA, USA), applying the one-way ANOVA, and pairwise differences were evaluated using Tukey’s multiple comparisons tests. *p* < 0.05 was considered statistically significant.

## 3. Results

### 3.1. LI-CNV Lesion Size Stabilized from Day 7 Post-Induction

**Figure 1:**
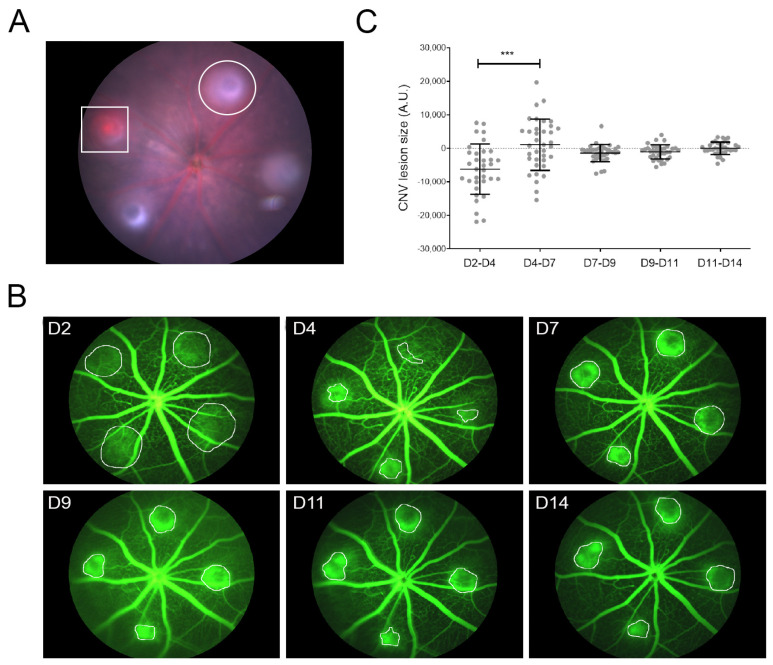
Analyses and lesion progression based on fluorescence angiography. (**A**) Example of an eye with four spots in the central retina, all equidistant from the optic nerve. The central retinal vessels were avoided to prevent severe bleeding. The circle indicates successful CNV lesions (vaporized bubble), and the square suggests la CNV with hemorrhage. (**B**) Representative fluorescent images of a CNV evolution in one eye. Fluorescence angiography was performed at 2, 4, 7, 9, 11, and 14 days post-laser to quantify the exudative areas of 4 lesions per eye. The quantification was performed by measuring the hyperfluorescent areas (circles are examples of the quantified areas). (**C**) Quantification analysis was performed by calculating the difference between lesion areas on consecutive days. Evolution of the exudates was statistically significant between the periods D2–D4 and D4–D7, being the latest period in which the lesions showed maximal growth (*** *p* < 0.0001; n = 35 lesions).

Argon laser irradiation successfully induced CNV in 92% of C57BL/6J male mice. The formation of a vaporization bubble indicated a successful burn, which correlated with Bruch’s membrane rupture (Figure 1A, circle). Hemorrhages appeared in some lesions (Figure 1A, square), indicating a retinal vessel puncture. In those cases, the lesion with the hemorrhage or the whole eye was excluded from the experiment. The size of the CNV lesions was first quantified in vivo using fluorescent angiography (Figure 1B,C). We monitored each lesion for 2 weeks by taking images of retinal vasculature on days 2, 4, 7, 9, 11, and 14. The relative growth of the lesions was calculated by comparing the lesion sizes between consecutive days (Figure 1C). On day 2, leakage appeared diffuse and weak, with unclear limits. At day 4, leaking areas were more defined and smaller, and between days 4 and 7, hyperfluorescent areas showed notable growth. After that, between days 7 and 14, the progression of the lesion areas was stable without any significant change.

**Figure 2:**
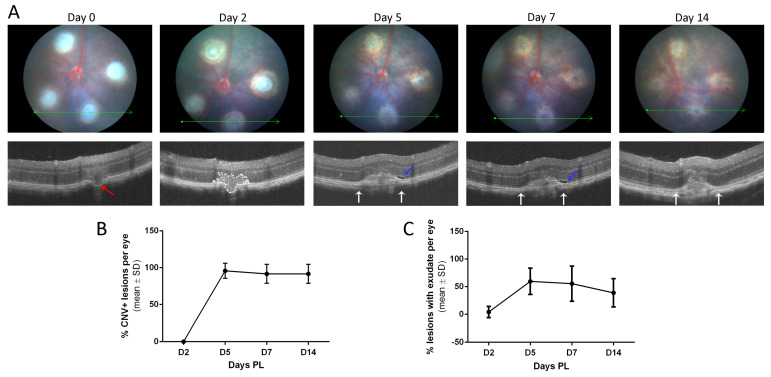
In vivo evaluation of CNV evolution based on OCT. (**A**) Fundus (above) and OCT images (below) were taken at 2, 5, 7, and 14 days post-laser to follow the progression of the exudative areas of 4 lesions per eye. Green lines depict the OCT B-scan images. Within the OCT images, the red arrow indicates the break of the Bruch’s membrane identified on day 0; the dotted line frames the butterfly-like hyper-reflective shape appearing by day 2; white arrows limit the hyper-reflective area corresponding to the CNV lesion detected by day 5; and blue arrows indicate the subretinal fluid associated with some lesions. (**B**) Graph shows that the percentage of CNV-positive (CNV+) lesions per eye that was 0% on day 2 increased to 96% on day 5. CNV+ lesions resemble inactive CNV and are characterized by heterogenous hyperfluorescence with late leakage in the subretinal region associated with pigment epithelium detachment. The margins of the hyperreflective lesion’s choroidal thickening, consisting of a flat lesion with a convex surface, were used to define lesion borders and quantify CNV+ lesions. (**C**) Graph shows the % of lesions with subretinal fluid. Subretinal fluid was present in 59% of lesions on day 5. Afterward, the presence of fluid experienced a slight decrease, as it was observed only in 39% of lesions on day 14. Data of both graphs are expressed as mean ± SD (n = 6 eyes).

The CNV lesion evolution was also analyzed through OCT. After laser photocoagulation, Bruch’s membrane rupture was detected in most lesions (Figure 2A, white arrow), whereas the photoreceptor layer over the lesion appeared hyper-reflective. On day 2, the injured areas showed the typical butterfly-like structure invading the ONL (Figure 2A, dotted white line), as previously described [39]. From day 5, a cellular mass corresponding to infiltrated cells was observed in the subretinal space in almost all of the lesions (Figure 2A, white arrows). Consequently, the ONL presented partial disorganization and degeneration in the CNV area and was consistent until day 14. At day 5, 96% of lesions developed neovascularization (Figure 2B). Moreover, in 56% of cases, a subretinal fluid accumulation appeared over or on the sides of the lesion (Figure 2A, blue arrows), indicating vascular leakage due to the weakness of the new vessels growing from the choroid. The presence of fluid detected was maximal at that point and slightly decreased to 38% on day 14 (Figure 2C). The findings show that the point of maximum growth of neovascular lesions occurs between days 5 and 7 in this model, indicating that the best periods for studying lesion growth/inhibition studies on acute lesions would be from day 0 to 4 and chronic lesion studies would be from day 7 onwards.

### 3.2. The LI-CNV Mouse Model Presents a Retinal Dysfunction in Lesioned Areas

**Figure 3:**
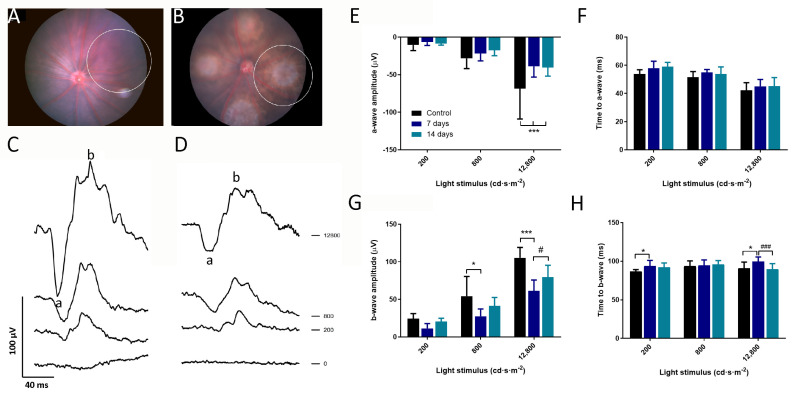
Changes in retinal function in LI-CNV lesions. (**A**,**B**) Representative images of color fundus photography corresponding to a control area (**A**) and an LI-CNV area (**B**) analyzed by using focal electroretinography (fERG). White circles indicate the area of the retina that was stimulated with the focal light beam. (**C**,**D**) Representative curve of fERG recordings indicating a- and b-waves at one light intensity in control (**C**) and LI-CNV (**D**) retinas. (**E**,**F**) a-wave and (**G**,**H**) b-wave amplitudes and implicit times at three different light intensities, comparing control areas (n = 7), LI-CNV at 7 days (n = 12), and LI-CNV at 14 days (n = 12). One week after laser photocoagulation, LI-CNV areas showed a statistically significant reduction in the a- and b-wave amplitudes compared to control areas and a significant delay in b-wave response. In contrast, on day 14, the b-wave amplitude and implicit time experienced a significant recovery compared to day 7. Graphs show mean ± S.D. (* *p* < 0.01; *** *p* < 0.0001 compared to the control; # *p* < 0.01; ### *p* < 0.0001 compared to day 7).

We analyzed the retinal response of LI-CNV and control areas by using focal ERG (fERG) (Figure 3A–D). This technique allows recording the functional response of a delimited area of the retina. Retinal responses were recorded on days 7 and 14 under scotopic conditions, using three different light stimuli intensities The a-wave amplitudes, corresponding to the photoreceptor’s function, were decreased on days 7 and 14 at high light intensity compared to controls (Figure 3E). In contrast, the implicit times were unaffected (Figure 3F). Similarly, the b-wave amplitude was significantly altered at day 7 by medium and high light intensities (Figure 3G), as lesions showed a response 32.7% lower than the one observed in control retinas. Interestingly, this functional impairment was less prominent on day 14, suggesting a partial restoration of visual response. Similarly, a delay of 5–10% in the implicit time in the b-wave was also found on day 7, both at low and high light stimuli, compared to control eyes, which significantly recovered on day 14 (Figure 3H). These data indicate a functional alteration in the outer and inner retinal layers more prominently at 7 days after laser photocoagulation, which correlates with the maximal growth of neovascularization in the subretinal space. Furthermore, we observed a partial recovery of the inner retinal function on day 14, which coincides with the stagnation of the progression of the neovascular lesion.

### 3.3. Neovascular Areas Are Maximal at 7 Days Post-Laser

**Figure 4:**
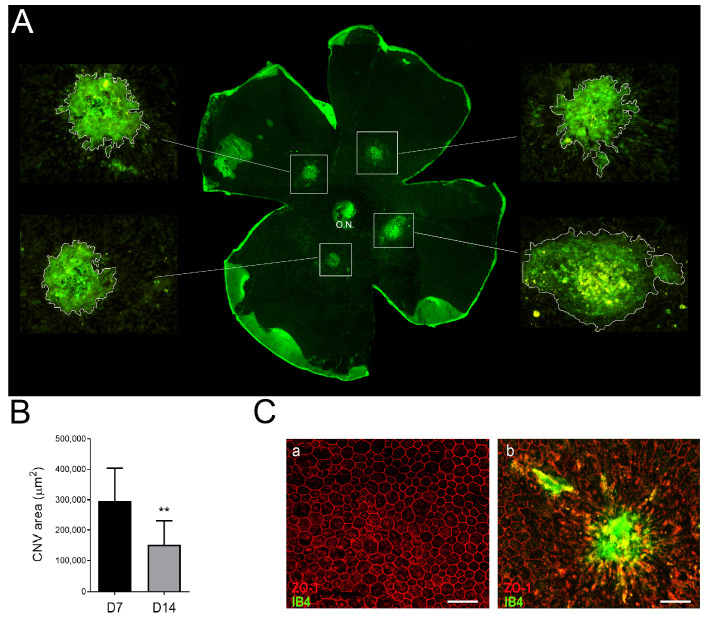
CNV quantification in flat-mounted eyecups. (**A**) Endothelial cells emerging from the choroid were stained with isolectin-B4 (IB4, in green) on flat-mounted eyecups (RPE-choroid-sclera) at day 7 post-laser. Higher-magnification CNV images of the boxed areas are shown. The optic nerve (O.N.) was also stained with IB4. (**B**) Quantification of IB4+ fluorescent-labeled neovascular areas using the software ImageJ. The mean area value on day 7 was significantly higher than that on day 14 (** *p* < 0.001, n = 10 eyes). (**C**) Immunostaining of RPE flat mounts (control (**a**) and LI-CNV (**b**)) with *zonula occludens* (ZO-1), a marker of RPE cells, and IB4. Scale bars: 50 µm.

We corroborated the presence of endothelial cells emerging from the choroid using the isolectin-B4 fluorescent staining in flat-mounted eyecup (Figure 4A). Quantification of LI-CNV lesions showed significantly larger size on day 7 compared to day 14 (Figure 4B), and the lesioned area presented a disruption of RPE cells and the infiltration of vascular endothelial cells (Figure 4C). These results concord with those observed through fluorescent angiography, OCT, and fERG, indicating a regression of the neovascularization process by days 7 to 14, which may correlate with the healing process of the lesions.

### 3.4. Laser Photocoagulation Induces a Pro-Inflammatory but Not a Pro-Angiogenic Response

**Figure 5:**
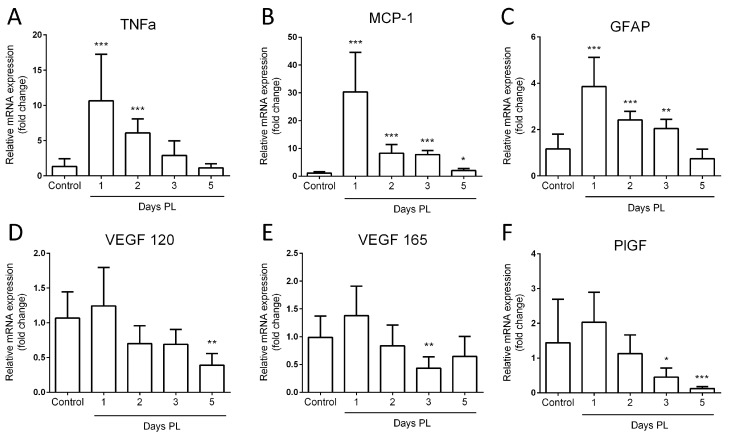
Gene expression of inflammatory and pro-angiogenic factors in the LI-CNV model. (**A**–**C**) mRNA expression of *Tnfα, Mcp-1*, and *Gfap* genes and (**D**–**F**) *Vegf 120, Vegf 165*, and *Pigf* genes was quantified by using real-time PCR. Relative mRNA expression was calculated using the B2M gene as the endogenous control. The statistical test applied was one-way ANOVA (* *p* < 0.01; ** *p* < 0.001; *** *p* < 0.0001; n = 10).

To elucidate the inflammatory and angiogenic status of the eyes in the LI-CNV mouse model, LI-CNV and control eyes were analyzed by using real-time PCR during the first 5 days corresponding to the acute stage when the main pathological process occurred. We quantified the changes in the mRNA levels of genes related to inflammation, such as the tumor necrosis factor-alpha (*TnfFα*), the monocyte chemoattractant protein 1 (*Mcp-1*), the glial fibrillary acidic protein (*Gfap*), and angiogenesis such as the vascular endothelial growth factor isoforms *Vegf-120* and *Vegf-165* and the phosphatidylinositol-glycan biosynthesis class F protein (*Pigf*). We found that *Tnfα*, *Mcp-1*, and *Gfap* analysis showed a statistically significant upregulation, reaching their maximal expression at day 1 and progressively returning to control levels until day 5 (Figure 5A–C). This expression trend may describe an acute inflammatory response caused by the laser burn that is not maintained over time. Similarly, after the initial upregulation, the expression levels of angiogenic factors such as *Vegf-120, Vegf-165*, and *Pigf* were significantly downregulated from day 3 to 5 (Figure 5D–F). These results indicate a global inhibition of the inflammation and pro-angiogenic responses in the whole posterior pole, possibly acting as a mechanism to counteract the local activation of endothelial growth in the lesioned areas.

### 3.5. Inflammation and Fibrosis Are Present in the LI-CNV Lesions

**Figure 6:**
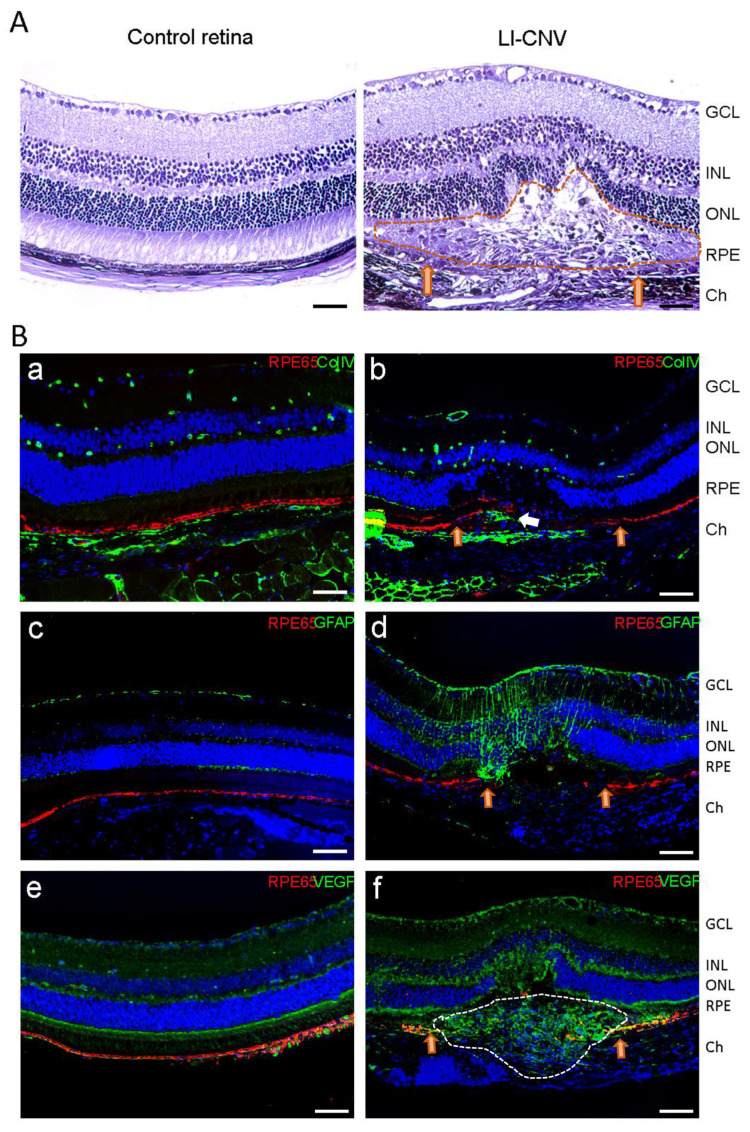
Immunofluorescence staining of CNV microsections. (**A**) Hematoxylin and eosin staining from control and LI-CNV retinal sections. CNV lesion with collagen visible and infiltrated cells in the sclera and within the lesion (dashed area). Orange arrows indicate RPE and Bruch’s membrane rupture. Scale bars: 50 µm. (**B**) (**a**,**c**,**e**) Control retinas and (**b**,**d**,**f**) LI-CNV lesions were double immunostained with RPE65 (a marker for RPE cells; RPE rupture is indicated with orange arrows in (**d**)), Collagen type IV (a marker for vascular endothelial cells and fibrotic mass of collagen within the CNV lesion (white arrow in (**b**)), GFAP (a marker for Müller glia activation), and VEGF-A (an angiogenesis factor; infiltrated endothelial cells is indicated with white dashed area in (**f**)). Nuclei were stained with DAPI. Scale bars: 50 µm. Abbreviations: Ch, choroid; GCL, ganglion cell layer; INL, inner nuclear layer; ONL, outer nuclear layer, RPE, retinal pigment epithelium.

We analyzed the overall status of CNV areas in the acute phase of lesion progression (3 days corresponding to the inflammatory response) compared to control retinas by histology and immunostaining of RPE (RPE65), the presence and localization of endothelial cells and the fibrosis in the subretinal space (Collagen IV), the glial activation (GFAP) and the expression of angiogenic factors (VEGF). As expected, most CNV lesions did not develop as organized retinal vascular structures but rather as disorganized endothelial growth in the subretinal space, together with infiltration of cells and fibrosis (Figure 6(A,Bb)). RPE65 staining revealed a discontinuity of the RPE layer due to the rupture of RPE-Bruch’s membrane unit in all the lesioned spots (Figure 6(Bb,d,f)), while it appeared intact in the control eyes (Figure 6(Ba,c,e)). Regarding inflammation, Müller cells’ end-feet showed intense staining of GFAP, and the radial processes also stained intensely throughout the inner and outer retina in the area surrounding the lesion (Figure 6(Bd)). In contrast, in control eyes, the expression of GFAP was lower, homogeneous, and mainly confined to the retinal ganglion cell layer (Figure 6(Bc)). This discontinuity in the RPE observed in the laser spots matched with an accumulation of Collagen IV- and VEGF-positive cells in the subretinal space, which indicated the presence of endothelial cells emerging from the choroid (Figure 6(Bf)), therefore confirming the initiation of the neovascular process and the initiation of fibrosis.

## 4. Discussion

The LI-CNV mouse model has been historically used to study and quantify neovascular progression and to monitor such progression upon different treatments. In our work, we contribute to the description of this model by deepening the retinal function upon laser photocoagulation. Our efforts have been directed at standardizing the methodology, optimizing the laser application with an image-guided laser system validated by other authors previously [51], the in vivo analysis of CNV evolution correlating the results obtained by using fluorescence angiography and OCT [45], the analysis of retinal function in the CNV areas applying the fERG, the vascular labeling and quantification, and the gene expression analysis.

Age-related macular degeneration, the leading cause of blindness in the Western world [52], is a complex chronic retina pathology that involves genetic and environmental risk factors and mainly affects the macula [53]. Because of these characteristics, generating a good animal model that fully mimics the pathology is complicated, especially because most of the species used in experimentation, except non-human primates, lack a macula. Despite this, several animal models recreate one or various pathological events present in AMD, either corresponding to its earliest or latest stages.

The evolution of the CNV in the LI-CNV model is highly influenced by the strain, age, and gender of the animals [7]. It is well known that genetic differences exist between the C57Bl/6J and 6N mouse strains, the last one carrying a spontaneous *rd8* mutation in the *Crb1* gene that produces a retinal degeneration phenotype [54,55]. Several mouse strains, including the widely used C57BL/6N strain, are susceptible to *Crb1* (Rd8) gene mutations. This mutation was found in all commercial sources of C57BL/6N, but not in the C57BL/6J substrain. Mice with the condition have ocular lesions, which could lead to a misinterpretation of the impact of a specific transgene or knockout. In this regard, we have found that the 6N strain produces bigger lesions [34,44] and suffers much more variability in the size and shape of lesions. Furthermore, C57BL/6J mice have a well-defined genetic background, making them an excellent choice for studies requiring genetic consistency. This consistency helps to reduce variability in experimental results, making it easier to compare results across studies. For this reason, most authors prefer to use the 6J strain [25,39,56]. However, one of the existing limitations in the literature is the lack of specification of the strain [33,35,57,58]. The age of the animals has been of great interest due to the differences observed in the progression of laser injuries [33,51], reporting more significant progression from 10 weeks of age. Regarding gender, it is not surprising that the pathogenesis, presentation, and causes of many diseases are gender dimorphic [59]. Although various authors recommend using females in the CNV induction experiments for their tendency to develop bigger neovascular areas [33,35], it is not fully clear the mechanism by which it happens, but it is described that older female mice exhibit more variable CNV areas [48,51]. We have detected more variability in female ERG recordings than in male mice (data not shown), indicating that estrogens may somehow interfere with the retinal pathological mechanism and function [60,61].

Laser injury to the RPE causes an initial wound-healing response that leads to the creation of neovascular lesions at the site of injury within a few days, and the area of these CNV lesions may be quantitated. The CNV evolution in the lasered spots was carefully controlled and validated both in vivo and in vitro. The confirmation of a break in Bruch’s membrane in terms of a so-called bubble formation by using color funduscopy was one standard in the exclusion criteria (e.g., hemorrhage after laser application) together with fused lesions and photocoagulation of the inner retina for excluding single laser spots or the whole eye. Only a few studies used OCT for confirmation [45], thus omitting to report whether a break could be confirmed. Similarly to Ragauskas et al. [45], fluorescent angiography and posterior hyperfluorescent area quantification allowed us to establish the maximal growth window between days 4 and 7, which was corroborated through OCT monitoring by quantifying extravasation. Moreover, this technique showed progressive stabilization from day 7. Moreover, our results obtained by using isolectin-B4 immunofluorescence in flat mounts, quantifying the presence of endothelial cells, pointed to a significant regression in the neovascular areas at 14 days. Moreover, the OCT analysis also reported a decrease in subretinal fluid events on day 14 compared to day 7, reinforcing the seventh-day theory as the maximum point of neovascularization and the subsequent regression and healing processes. In this regard, we have found discrepancies in the bibliography, with some authors agreeing that there are no significant differences between 7 and 14 days post-laser [48,62], others reporting larger lesions sized at day 14 and a later regression [33], and others observing a dissipation of the subretinal fluid accumulations from day 7 onward and a statistically significant decline in the lesion sizes from days 7 to 28 [39].

Our report describes the use of the focal ERG technique for the first time to record the ERG signal of small retinal CNV areas. We could measure the local retinal responses in the vicinity of the CNV lesion and assess how the neovascularization impacts retinal function. We compared the ERG waves’ amplitudes and implicit times at different light stimuli in control and lesioned retinas at days 7 and 14 days. We observed a significant impairment of the functional response (both a- and b-waves) on day 7. Interestingly, only the b-wave function showed a substantial recovery on day 14. The decrease and slowdown of the ERG a-wave in the lasered spots clearly indicate the degeneration of photoreceptors in the lesions. In contrast, the partial recovery of a b-wave may indicate that altered kinetics of neural adaptation in these areas, most probably produced by the RPE injury and the subsequent retinal detachment and inflammation, was partially restored at day 14. These data strengthen the evidence of retinal function fluctuation in the impaired retinal areas by a laser BM break, correlated with the evolution of neovascular processes. Similarly, Caicedo et al. [57] pointed to an evident full-field ERG impairment in the treated eyes 7 days after laser injury, although the b-wave amplitude reduction was maintained in the posterior weeks. In our hands, the ERG recovery goes according to the CNV analyses, which corroborates the initiation of a regression and wound-healing process during the second week after the laser.

The inflammatory factors analyzed, mostly *Tnfα* and *Mcp-1*, underwent upregulation, with a peak in the first hours after laser burn and a progressive return to the basal levels at day 5, mimicking a typical acute inflammatory process. In this sense, other authors have reported larger periods of an inflammatory response [63,64], and the contribution of infiltrated macrophages and local glia activation to the CNV model [56,64,65,66,67] is widely accepted. Although it is firmly established that VEGF is the principal mediator of ocular angiogenesis and various authors affirmed a strong upregulation of *Vegf* mRNA and protein in the lasered eyes [65,66,67,68], in our case, we did not find a significant variation in *Vegf* levels in the whole posterior eyecups in the first three days after laser but a slight inhibition from day 5. Instead, a high expression of VEGF was detected histochemically, mainly in the endothelial cells emerging from the choroid, indicating that the angiogenic response may not be a global response in the eye but a localized expression by the growing vascular endothelium. This observation is supported by the results of André et al. [63], who indicated a localized VEGF expression surrounding the lesioned area in RPE/choroid flat mounts.

The main flaw in the rodent LI-CNV model is that it cannot accurately reproduce the intricate series of circumstances that result in CNV in AMD. Instead of being the outcome of long-term senescent degeneration and chronic inflammation, it is a model of acute damage and inflammation. Since laser coagulation significantly damages the overlaying neuronal retina to a more significant extent than is typical of human AMD, there are also anatomical differences. The mouse eye is smaller than the human eye and lacks a macula. Although it is the gold standard model for wet AMD, laser-induced CNV is prone to important investigator-dependent factors that must be reduced and standardized to ensure consistency across research groups. CNV lesions are produced by laser photocoagulation, and the quantification techniques utilized can impact experimental results in addition to altering the photocoagulation process itself. Researchers have greatly improved these procedures, greatly enhancing the reproducibility of illness induction and measurement. Additionally, biological factors, including housing conditions and diet (mostly lipid composition) [69,70], might also influence the course and severity of laser-induced CNV. Nevertheless, the LI-CNV mouse model has significantly contributed to our current comprehension of the etiology of CNV.

Despite its limitations, the laser-induced CNV model is one of the most effective tools for researching the crippling pathophysiology of neovascular AMD. Its application has aided in the development of many of the AMD medicines that are currently accessible, as well as deepening our understanding of the etiology of AMD. This model can only measure CNV lesion size after experimentally generated injury, implying we can only analyze the impact of genetic or pharmacologic interventions on CNV growth, not factors that influence CNV lesion generation. It will be critical to evaluate how pharmacologic targeting of inflammasomes could be used for innovative AMD treatments. Although angiogenesis and inflammation may appear to be two distinct processes, they are actually tightly related, particularly when angiogenesis occurs in adult organisms and is frequently accompanied by inflammation [71]. Recent studies suggest that several anti-inflammatory approaches such as Resveratrol/Metformi [72], Lycopene [73], cerium oxide nanoparticles [44], hydrogen gas [74], Shikonin [75], fursultiamine [76], Andrographolide [77], 5′-glucuronyl azidothymidine [78], and Pirfenidone [79], among others, can reduce laser-induced CNV formation in mice in a proof-of-principle method.

In turn, hypoxia and immune cell infiltration are characteristics of inflamed tissues, which will cause an increase in cellular and molecular pathways that control angiogenesis [80]. Because of the role of inflammation in neovascular AMD, conventional therapy focusing solely on reducing angiogenesis may not be the best option. Most likely, inflammation does not cause AMD in the first place, but it does manifest in early AMD pathology, which may help to explain why anti-inflammatory drugs work well as preventive or supplemental treatments when combined with anti-VEGF therapy, photodynamic therapy, or both [81]. Nonsteroidal anti-inflammatory drugs and steroids have been studied for synergistic or additive effects in clinics. Dexamethasone, ketorolac eyedrops, or triamcinolone acetonide reduced the requirement for adjunctive ranibizumab medication and demonstrated satisfactory tolerability in patients with neovascular AMD [82,83,84,85,86].

## 5. Conclusions

Our results suggest that the progression of our LI-CNV model can be foreseen in two phases. First, an initial phase involves an acute inflammatory response during the first 3 days after laser photocoagulation with overexpression of inflammatory factors such as *Gfap*, *McpP-1*, and *Tnfα* and a rapid evolution of lesion morphology and appearance, followed by an intermediate phase from days 3 to 5, a decrease in inflammation, and the intrusion of endothelial cells overexpressing VEGF, which becomes more prominent. Then, an exponential phase from days 5 to 7 is characterized by a maximal growth of the neovascular areas and a retinal function alteration in the adjacent retina. Finally, a second phase lasts between days 7 to 14, during which the injured retinas lose their inflamed status, neovascular lesions reduce their growth with some levels of wound healing, and the adjacent retina restores its normal function, reaching a stabilization phase. We believe that these are the reporting of parameters essential for the validity, consistency, and reproducibility of experimental CNV research.

## Data Availability

Not applicable.
